# Strategies to Address the Complex Challenge of Improving Regional and Remote Children’s Fruit and Vegetable Consumption

**DOI:** 10.3390/nu10111603

**Published:** 2018-11-01

**Authors:** Stephanie L. Godrich, Christina R. Davies, Jill Darby, Amanda Devine

**Affiliations:** 1 School of Medical and Health Sciences, Edith Cowan University, 270 Joondalup Drive, Joondalup, Perth 6027, Australia; j.darby@ecu.edu.au (J.D.); a.devine@ecu.edu.au (A.D.); 2 School of Population Health, University of Western Australia, Crawley 6009, Australia; christina.davies@uwa.edu.au

**Keywords:** fruit and vegetables, rural children, Social Cognitive Theory

## Abstract

Fruit and vegetables (F&V) are imperative for good health, yet less than one per cent of Australian children consume these food groups in sufficient quantities. As guided by Social Cognitive Theory (SCT), this paper aimed to: (i) understand key informant perspectives of the amount, types and quality of F&V consumed by rural and remote Western Australian (WA) children; and, (ii) determine strategies that could increase F&V consumption among rural and remote WA children. This qualitative study included 20 semi-structured interviews with health, school/youth and food supply workers, focusing on topics including: quantity and type of F&V consumed and strategies to increase children’s consumption. A thematic analysis was conducted using NVivo qualitative data analysis software (Version 10, 2014. QSR International Pty Ltd., Doncaster, Victoria, Australia). Key informants reported children consumed energy-dense nutrient-poor foods in place of F&V. Strategy themes included: using relevant motivators for children to increase their preference for F&V (i.e., gaming approach, SCT construct of ‘expectations’); empowering community-driven initiatives (i.e., kitchen gardens, SCT construct of ‘environment’); increasing food literacy across settings (i.e., food literacy skills, SCT construct of ‘behavioural capacity’); developing salient messages and cooking tips that resonate with parents (i.e., parent newsletters, SCT construct of ‘self-control’); increasing F&V availability, safety, and convenience (i.e., school provision); and, considering the impact of role models that extend beyond the family (i.e., relatable role models, SCT construct of ‘observational learning’). Overall, a comprehensive strategy that incorporates relevant motivators for children and families, supports local initiatives, reinforces the range of role models that are involved with children and creates healthier environments, is required to increase F&V consumption among children.

## 1. Introduction

Fruit and vegetables (F&V) are essential to good health, containing a vast array of indispensable nutrients and bioactive compounds, such as vitamins, minerals, electrolytes, dietary fibre, protein, and phytochemicals [1]. F&V are known for their high concentrations of specific phytochemical or ‘non nutrient’ properties, such as carotenoids [2], flavonoids [3,4], phytoestrogens [3], and terpenes. In addition, F&V have a relatively low energy density and a high water concentration [5].

F&V provide a myriad of health benefits, including a reduced risk of non-communicable diseases [6]. Such diseases include cancer through hormone-inhibiting roles [7,8], Type II diabetes [9], and a probable link with reduction in obesity [2,7,9]. Vegetables have been shown to have a cardio protective effect, reducing the risk of cardiovascular disease and stroke [10], namely due to containing a plethora of antioxidant compounds that repair cell damage due to oxidation [3]. The role of nitrates in vascular tone and integrity have also been recently reported [11,12,13,14]. Further, F&V have been recently shown to reduce risk of falls and fractures that are related to superior muscular strength (grip strength) and physical function in those with higher intakes and a more diverse range of vegetable intake [15]. Moreover, the benefits of vegetables on bone health include the action of nutrients, such as vitamin K, magnesium, phytoestrogens, and other active compounds on improved bone metabolism [16,17]. Given the wide-ranging health benefits that are provided by F&V, adequate consumption in childhood is critically important. This is especially important, given child eating habits track into adulthood [5].

Despite these health benefits and evidence associating poor childhood intake of F&V with subsequent low adult consumption [18], many Australian children are consuming insufficient F&V [19]. In Australia, “sufficient” consumption of F&V is two serves of fruit (i.e., two medium pieces) and five serves of vegetables for children aged 9–11 years and females aged 12–13 years [20], where one serve is equivalent to one cup of salad or half a cup of cooked vegetables [20,21]. For boys aged 12–13 years, the recommended vegetable intake is 5.5 serves [20].

National data estimates that less than one per cent of Australian children aged 9–13 years are consuming vegetables in sufficient quantities for good health [22]. A higher proportion of Australian children reportedly met fruit guidelines, with 45% of 9–11 year olds and 34% of 12–13 year old children achieving the recommended fruit serves per day [22]. Consumption of F&V is higher across Western Australia (WA), with statewide surveillance data reporting 8.8% of children met vegetable guidelines and 64% met fruit guidelines [23]. Recent research that was conducted specifically in rural and remote WA reported a slightly higher proportion of children met guidelines, with 65.8% meeting fruit and 15.4% meeting vegetable guidelines, respectively. While in rural Victoria, 97% of children aged 6–12 years consumed adequate fruit and 12% consumed adequate vegetables [24]. To date, comprehensive data outlining the difference in F&V consumption between urban, rural, and remote children is lacking, due to limitations that are associated with availability of data at a local level [25].

With respect to the classification of rural and remote Australia, the Australian Statistical Geography Standard Remoteness Structure classifies locations in relation to their access to services [26]. Covering one-third of Australia’s land mass, WA spans a vast 2.5 million square kilometres [27], with much of this state being classified as remote.

National data has demonstrated that people living in remote locations are more likely to be overweight or obese than their urban counterparts, with reduced health service provision purported to be a contributing factor [25]. A number of barriers to F&V consumption have been postulated, including price, promotion, low availability, poor quality, location of outlets, and taste preferences [28]. These are particularly magnified in rural and remote communities where food availability and access issues are heightened, such as through transport disruptions [29,30]. As poor diet, such as low consumption of F&V, is a contributing factor towards overweight and obesity [6], and given the aforementioned barriers to F&V consumption in rural and remote locations, the consumption levels could be estimated to be lower in rural and remote areas.

To overcome barriers to F&V consumption among children, a number of strategies have been proposed internationally and nationally, such as a settings approach where core foods are provided or nutrition education delivered in early years and school settings [5,28]. School-based interventions have achieved moderate increases in children’s intake of F&V [18]. To increase impact, suggested approaches have included extending school-based activities to the home environment, whereby parents are engaged through activities delivered by teachers and health professionals [31,32]. To increase relevance for child audiences, a focus on fun has been recommended as more salient [31] and it should be a key strategy adopted in initiatives. Incentives and rewards should also be incorporated [5], with children provided the freedom to make personal food choices [31]. In addition, goal setting, role playing, and critical thinking are advocated for [5]. Overall, longer-term, higher intensity interventions with multiple strategies have been demonstrated to be most effective in increasing children’s F&V consumption [18,33].

However, there is limited evidence for successful strategies that specifically target rural and remote populations. Gaps in the literature include an understanding of high-risk and disadvantaged groups’ needs, such as those living in rural and remote areas [34]. Recent research [35] has highlighted the differences in healthy food availability, access and use in rural and remote locations, and the associated impact on children’s F&V consumption [33]. Therefore, these differences must be front and centre when developing strategies to increase consumption among rural and remote children.

One framework that provides a useful context in which to situate strategies to increase F&V consumption is the Social Cognitive Theory (SCT) [36]. The SCT comprises nine constructs and considers the impact of personal factors, such as food preferences, expectations of consuming particular foods, knowledge, and self-efficacy on a behavior, such as F&V consumption. In addition, the impact of environmental factors, such as availability, accessibility, parents, peers, and the media, on the behavior, are also considered [5]. Previous research has argued this theory is ideal for the examination of the impact of interventions on children’s F&V intake [5].

Given there are a number of gaps in the current evidence base relating to the increased support required for rural and remote children to consume adequate F&V, this study aimed to extend existing literature to understand potential strategies to increase rural and remote children’s F&V consumption. The objectives of this study were to: (i) understand key informant perspectives of the amount, types, and quality of F&V consumed by rural and remote WA children; and; (ii) determine strategies that could increase F&V consumption among rural and remote WA children, using a SCT focus.

## 2. Materials and Methods

### 2.1. Design and Sampling

The study methodology is reported elsewhere [30]; a summary is provided below. ‘Key informants’ were the selected participant group for this qualitative exploratory research study. These worker types included health workers (e.g., nutritionists, dietitians, health promotion officers), school/youth workers (e.g., school principals, youth, and family workers who worked with both primary school age children—junior, previously years 1–7 and currently years 1–6 in Western Australia, and secondary school age children—senior, previously years 8–12 and currently years 7–12) [28]. Food supply workers (e.g., included store managers and farmers’ market managers). These key informants were involved with children’s F&V consumption, such as through a breakfast or after school feeding program, delivery of health promotion programs, or owned/managed a food outlet where children and/or their parents shopped. A database was developed using contact details from professional networks (health and school/youth workers) and Google searches using the keywords “supermarket” and the town name (food supply workers).

WA is categorised by the state government into nine regions (i.e., Peel, South West, Great Southern, Wheatbelt, Goldfields-Esperance, Mid West, Gascoyne, Pilbara, Kimberley regions) [37]. It is also classified into degrees of remoteness, according to the Australian Statistical Geography Standard Remoteness Areas [26]. Key informant recruitment was conducted to ensure diversity across WA regions, degrees of remoteness, and worker type, through the development of a spreadsheet with these criteria. A total of eight out of the nine WA regions invited were represented in this study. Where possible, sampling in this study was closely representative of the population density across WA regions [37] and degrees of remoteness. Key informant locations also ranged in Socio-Economic Index for Areas Index of Relative Socio-Economic Disadvantage decile [30,38] to ensure that areas of varying socio-economic disadvantage were included.

### 2.2. Data Collection

Thirty potential interviewees were invited to participate in this study and were provided with an information letter and consent form. Twenty respondents provided written consent (67% response rate) and took part in a semi-structured interview, conducted either by telephone (*n* = 16) or face-to-face (*n* = 4). The interview guide was compiled by the research team and contained questions related to quantity and type of F&V consumed (i.e., “What are your thoughts around the amount of F&V kids are eating in the areas you live/work in?”); barriers to and enablers of F&V consumption among children (i.e., “What types of things make it hard for children to eat F&V in the areas you live/work in?”); motivators of intake (i.e., “What do you think are the biggest motivators for F&V consumption among WA kids?”); and, strategies (existing or proposed) to increase children’s consumption (i.e., “What successful strategies have you seen, where you work?”).

One pilot interview was conducted, with minor changes made before use in the wider study.

### 2.3. Data Analysis

Key informant interviews were transcribed verbatim and imported into QSR Nvivo 10 (NVivo qualitative data analysis Software; Version 10, 2014. QSR International Pty Ltd., Doncaster, Victoria, Australia) A thematic analysis was conducted using techniques, such as template analysis and parallel coding [39]. In addition, word clouds and matrix-coding queries provided further insight into themes determined. Inductive, data-driven codes were created as new themes were identified. Three team members verified coding of statements by co-listening to audio recordings and cross-checking the NVivo coding. Documents including a research journal and summary of work conducted outlined codes created with exemplar quotes, key themes, and other analyses undertaken in NVivo. Saturation was confirmed at 20 interviews when the team confirmed that there were no new themes or concepts that had been identified [30,40].

### 2.4. Ethical Approvals

All of the subjects gave their informed consent for inclusion before they participated in the study. The study was conducted in accordance with the Declaration of Helsinki. Ethics was obtained from the Edith Cowan University Human Research Ethics Committee.

## 3. Results

### 3.1. Demographics

Interviewee demographics included 16 females and four males; 12 key informants reported on regional WA, while eight reported on remote WA. A total of eight respondents were health workers (i.e., dietitian, nutritionist, health promotion officer), six were school and/or youth workers (i.e., school principal), and six were food supply workers (i.e., farmers’ market manager). Key informant demographics have been tabulated in a previous publication [30]. The results below have been grouped into suggested strategies (existing or proposed) to increase children’s F&V consumption. Exemplar quotes for each strategy have been included (Table 1).

### 3.2. Perceptions of Consumption

Key informant perspectives with respect to the quantities of F&V consumed by children included comments such as “not enough” (Food Supply Worker, Female, Regional), “low compared to the amount of ‘extras’ that are consumed” (Health Worker, Female, Regional), “without the schools, that would be much more of an issue” (Health Worker, Male, Remote) and “Relatively lower than what is recommended” (Health Worker, Female. Regional). Among the 16 coded statements about F&V quantities consumed, some informants reported observing parents and children buying energy dense nutrient-poor foods, such as pies and sausage rolls, or high-carbohydrate filling foods, as opposed to F&V. A view shared by a number of participants was that adequate fruit consumption was more common than adequate vegetable consumption, which key informants perceived as being poor amongst most children. In particular, children’s lunchboxes were seen to be more fruit-focused, with children “going to school without any vegetables in their lunchboxes at all” (Health Worker, Female, Regional). Perceptions of F&V types that were consumed included that they were dependent on the availability within a food outlet. Other perspectives were that school canteens were providing locally-produced F&V and that school based activities supported and promoted access to them.

### 3.3. Strategies to Increase Consumption

#### 3.3.1. Strategy One: Use Relevant Motivators for Children to Increase Their Preference for Fruit and Vegetables

Based on their experiences and perceptions, Food Supply and Health Workers indicated that focusing messaging on the impact of healthy eating on sporting performance might be an effective strategy to encourage children to consume more F&V. The perspectives of School/Youth Workers focused on education about healthy food choices using a strengths-based approach, i.e., “I certainly think it makes a massive difference if they have had education around healthy choices at some stage” (School/Youth Worker, Female, Regional).

Many children were believed to understand that eating F&V kept them healthy, however, messages needed to extend beyond this due to the lack of consideration many children placed on long-term health implications that are associated with suboptimal consumption. Utilising a different perspective, such as reinforcing the benefits of being strong, fit and promoting a healthy body image, was an important consideration when promoting healthy eating. This was thought to be especially effective among teenage boys, where the strengths-based focus was on being fit. Insights included the importance of reinforcing fun, creative and experiential learning around the growing and preparation of healthy food to positively influence children’s attitude to F&V. According to interviewees, children and young people sought independent food choice, and thus focusing on encouraging choice of healthy foods was important. One informant discussed their approach, which focused on promoting the enjoyment of tasting fresh F&V. This was also suggested to be done in settings, such as community garden plots. Further, promoting F&V in other settings, such as through family-friendly arts events, was another avenue for promotion. Other strategies included presenting information about the negative health consequences of low F&V consumption in movies compiled in an entertaining way.

#### 3.3.2. Strategy Two: Empower Local Community-Driven Initiatives

Key informants discussed the importance of local community spaces and activities that promoted F&V consumption, particularly for rural and remote populations. These included community gardens, communal cooking facilities, and sporting events. Further, participants in this study reported the creation and display of healthy eating posters in food outlets and community cookbooks were successful in increasing F&V consumption. Health service providers were encouraged to support existing or the start-up of local initiatives, which had a more significant and longer lasting impact. For example, local community gardens or kitchens, which could also be used to upskill participants in short courses, such as food hygiene. In addition, practitioners embraced groups’ evolving interests in healthy eating skills, such as a group formed initially to focus on exercise, evolved to focus on food label reading. Key informants emphasized the importance of a place-based approach with respect to supporting local preferences and needs, as well as showcasing local role models who were often better placed to upskill fellow community members, rather than relying on service delivery or resource creation from ‘outsiders’, i.e., “In terms of the women making healthy eating posters…I just think local stuff has a huge impact as people are obviously learning through the making of the stuff as well as with the sharing” (School/Youth Worker, Female, Remote).

#### 3.3.3. Strategy Three: Increase Food Literacy Education across Settings

A number of key informants were concerned that inequitable health education and support services resulted in certain life stages being disadvantaged. For example, the early years sector, an area that is cited as a critical life stage in which lifelong healthy habits could be initiated, and subsequently consolidated upon entry into the school setting. A number of stakeholders believed that school education could be the catalyst for health-promoting behaviours across the lifespan, i.e., “You don’t always have a break through but sometimes you have a spark that goes and they will keep that with them for the rest of their lives” (Food Supply Worker, Female, Regional). The application of knowledge gained through curriculum activities was reportedly supported through experiential programs combining food production and preparation, with produce tasting a critical element. One stakeholder reiterated the importance of F&V provision through the school setting, such as through morning recess. Two stakeholders recounted the success of ‘meet the farmer’ or farm visits that had been held with the local school children. This approach to education reportedly increased awareness of the food system.

#### 3.3.4. Strategy Four: Develop Salient Key Messages and Cooking Tips That Resonate with Parents

Practical strategies for parents to incorporate F&V into their family’s diet were suggested, including disguising these foods into meals and snacks, i.e., “Making kebabs out of fruits, using the dips, putting pureed vegetables into things, like bolognaise sauces. Making rissoles with grated vegetables so they can disguise them to a degree” (Health Worker, Female, Regional). Other strategies included the provision of easily accessible produce, such as having chopped F&V within easy reach on the kitchen bench when children were hungry. Another informant suggested allowing the child to choose a novel fruit or vegetable each week, and making a new, in-season fruit the ‘treat’. Exposure to new preparation methods and recipes through family, friends, local stores or the school environment was also recommended.

#### 3.3.5. Strategy Five: Increase Fruit and Vegetable Availability, Safety and Convenience

One informant recommended interactive strategies, such as consumer engagement through food tastings in-store, or pairing recipes next to ingredients that could be used to make the meal. One participant reflected on her own practice to support healthy eating in her store, through the preparation of F&V snack trays. Others believed that offering mid-week farmers’ markets or increasing the percentage of F&V within takeaway outlets would improve food availability in rural areas. i.e., “Get the fast takeaways with a greater percentage of vegetables and fruit in there and it may become more popular” (School/Youth Worker, Female, Remote). Another informant criticized the significant proportion of high quality produce that is exported to other countries, and commented that Australia should be prioritising the best quality F&V for local outlets instead. A number of informants underscored the importance of schools providing nutritious food to children, particularly with an emphasis on F&V.

#### 3.3.6. Strategy Six: Consider the Impact of Role Models that Extend Beyond the Family

Key informants discussed the importance of role models outside of the family unit. For example, family friends who may have prepared meals using unfamiliar F&V, cooking methods, or food preparation skills, i.e., “being introduced to foods you might not have at home, just in different ways” (Health Worker, Female, Regional). This approach was suggested to improve their amenability to tasting less familiar foods. Elite athletes were admired in rural and remote areas, with their influence apparent when they participated in school communication projects.

## 4. Discussion

The objectives of this research were to understand key informant perspectives of amounts, types and the quality of F&V consumed by children; perceived motivating factors for consumption; and, effective strategies for increasing children’s F&V consumption. Key findings included increasing children’s preference for F&V through the utilisation of strategies using relevant motivators, empowering local community-driven initiatives, increasing food literacy in early childhood and school settings, developing salient messages for parents, increasing availability, safety, and convenience of F&V in outlets, and considering the impact of role models that extend beyond the family.

Key informants in this study reflected on their experiences that were associated with children’s F&V consumption. These included that fruit consumption was higher than vegetable consumption and substantially and positively influenced by the school environment. This was previously reported in a study conducted in rural and remote WA where schools were thought to facilitate consumption through breakfast or lunch programs [28]. Previous research has also reported F&V in food outlets is inconsistent in regards to availability, quality, and promotion [30].

This study highlighted the importance of using relevant motivators to increase children’s food preference for F&V. Interviewees (health, school/youth, and food supply workers) reflected on their own successful experiences where they had utilised more personalized strategies to increase F&V consumption. Examples included reinforcing the benefits of being strong, fit, and promoting a healthy body image. The use of fun, creative, and experiential learning was found to positively influence children’s attitude to F&V. This supports our previous work that found attitude was a key driver of F&V consumption among WA children [28]. In their study of children in rural New Zealand, Dresler et al. recommended child-focused F&V campaigns should utilize the compelling techniques that are used by large companies to emphasise fun, energy, and colour of the produce [41]. In addition, cartoons depicting characters choosing healthy food have demonstrated a shift in children’s food choices to healthier options [42]. An after-school nutrition intervention that was underpinned by the SCT with the aim of improved dietary self-efficacy, included activities focusing on exposure to healthy food, taste testing, and the link between diet and physical activity [43]. Younger children (aged 5–10 years) demonstrated a significant improvement in dietary self-efficacy because of the intervention [43]. However, the intervention had no effect on older children and adolescents [43]. Family-friendly arts events were suggested in this study as an avenue for message delivery. WA evidence corroborated this suggestion, with a significant association between nutrition messages and arts engagement being found [44].

Empowerment of local community-driven initiatives was a key theme in the current study. The focus on local community spaces promoting F&V consumption, such as community gardens, communal cooking facilities, and sporting events, were reportedly highly successful vehicles for promoting consumption of these food groups, based on key informant perceptions and experiences. Further, the creation of health promotion posters displayed in food outlets and cookbooks were reported upon favourably, with particular value being placed on the development of these initiatives by people within the community. Locally developed initiatives reportedly had a more significant and longer lasting impact than those “brought in” by outside service providers. This is of particular importance in rural areas, where service providers sometimes facilitate unsustainable programs that do not consider the local context [28]. Interest in the notion of ‘co design’ has increased in recent years, with benefits of collaborative design of projects reportedly including more meaningful service provision and increased consumer satisfaction [45]. Community support, such as through the provision of community knowledge and local leadership, is central to successful public health nutrition interventions. Community organisation and action can ensure communities being about health changes that they identify as priorities [46]. Extending this further, the development of social capital by initiatives is a vital success factor; those initiatives that facilitate collective social capital increase community competence in addressing issues [47,48], and ultimately, improve their health [46].

Increasing food literacy across key settings such as early years settings, schools, and workplaces emerged as an important theme in this research. Some informants commented that inadequate service provision resulted in certain life stages missing out on food literacy education. Curriculum activities which focused on experiential learning through food production, preparation and tasting were celebrated. Farm visits were also well-regarded as a powerful educational experience, and they are a unique vehicle for education in rural areas where there is a closer connection to food producers [28]. Food literacy education can increase children’s understanding of how to choose healthy foods, not only for themselves, but their family [49]. In the early years sector, a recent umbrella review that examined 12 systematic reviews of 101 studies reported that healthy eating approaches embedded within services improved healthy eating in children aged 2–5 years [32]. The review found the most successful interventions were delivered by nutrition and health experts, targeted both the centre environment and the individual, included a mix of strategies that were addressed through the curricula, food and the physical environment, staff practice and policy, and engagement with parents [32]. Within a school setting, specific strategies have included designated F&V breaks or activities, such as taste testing [33] and using core food groups for curriculum activities instead of discretionary foods [50]. A WA school-based F&V program found that significantly more children brought vegetables to school as a result of targeted curriculum and parent resources [51]. However, many children have been reported to possess limited food literacy skills, including poor awareness of what constitutes healthy food choices and limited preparation skills. School programs should be linked with the curriculum, utilise media [41], and include experiential learning to enhance skills [28]. The development and possession of food skills among children and adolescents translates to favourable health behaviours, and thus, improved dietary quality [52] and has been shown to decrease children’s requests for energy dense nutrient poor foods, which contribute to obesity [52]. Further, food literacy among children and adolescents can increase their beliefs regarding the “social value of food, food system issues and relationships between the food system and environmental sustainability” [49].

It is critical that health promotion projects aiming to improve healthy food consumption among children, include salient key messages and cooking tips that resonate with household gatekeepers; parents. Parental nutrition knowledge and cooking skills are essential factors to consider in the investigation of barriers and enablers of children’s F&V consumption, such that specific skills in planning, decision-making, purchasing, preparing, and understanding the impact of food on health are required to meet dietary needs [53]. Adults have been suggested to lack important food purchasing and preparation skills, which reduced their children’s consumption of F&V [54,55].

Messages to improve food planning, purchasing, and preparation should be clear, specific, and focus on behavior change [56]. Messaging should also focus on increasing F&V in the home environment, with resources such as newsletters utilising increasing parental engagement and understanding [33]. Importantly, messages must consider the rural or remote context in which they are being delivered, such as tailoring messaging about F&V purchasing to reflect availability, cost, and quality considerations [56].

The current research emphasized the importance of increasing fruit and vegetables availability and convenience. Strategies that were recalled by informants included food tastings in-store, or presenting ingredients to comprise a meal in the same location of a store. One store owner prepared F&V snack trays to prompt increased purchasing, while other informants recommended offering mid-week farmers’ markets or increasing the percentage of F&V within takeaway outlets as strategies to increase the availability of these core food groups. These findings concur with other research, which has emphasised the importance of changing environments to include more F&V. Recent WA evidence has cited the limited availability of fresh vegetables in rural areas as problematic to support interventions, in comparison to urban environments [35]. Evidence highlighted the success that is associated with increasing provision of a range of vegetables, improving their presentation, changing the order in which they were served or changing their location. These actions resulted in increased selection and consumption of these foods in children [1]. Further, other researchers have demonstrated that provision through settings such as schools can increase consumption of fruit by 0.27 servings per day and combined F&V servings of 0.28 servings per day [57]. The inclusion of salad bars in U.S schools have encouraged increased F&V consumption among children and youth, with particular success being observed in increased fruit consumption. Further, including locally-produced F&V in the salad bar coupled with nutrition education, could be an additional strategy [58]. Moreover, policy-level interventions, such as subsidised vegetables in rural areas, have been requested [35].

The important influence of role models that extend beyond the family was highlighted in this study. Key informants underscored the importance of role models, including family friends and elite athletes. Previous evidence has concurred that role modeling of health-promoting behaviours, such as consuming F&V, has a positive correlation with intake of these foods among children. These results suggest that children are conscious of their parents’ participation in these behaviours, which, in turn, have a positive impact on their own behaviour [59]. Previous WA evidence suggests that parents in rural locations may be more likely to support F&V interventions in school settings, which suggests this could be a core area to focus on in future interventions. The same study also found more rural participants recommended resources to increase child and parental knowledge about vegetables, in comparison to urban locations [35]. Recommendations arising from this research support previous evidence, which includes a multi-pronged approach to increase F&V intake among children.

Using relevant motivators that are salient to children align with the ‘expectations’ construct of the SCT. While skill development and taste testing [5] align with the behavioural capacity element, self-control, such as through role modelling, has been shown to be an effective strategy to increase consumption [5]. This latter activity can be adapted across settings, such as the early years settings and transition into the school years.

Strategies to empower community-based initiatives to focus on the environmental construct of the SCT need to be considered. These include increasing availability of F&V across settings and supporting initiatives like community gardens in locations close to early years services and schools.

When supporting food literacy development across settings, focusing on the behavioural capacity construct of the SCT, a focus could include skill development through the provision of recipes and an educational component. Again, these initiatives are translatable in settings such as childcare, school, food outlets, as well as in the home environment.

Developing salient messages for parents, through a focus of the self-control construct of the SCT, could incorporate cooking tips in local newsletters or developing collaborative cook books with parents [5]. This could also develop self-efficacy, an important element to increase F&V consumption through targeted messaging [5].

Increasing F&V availability, through the environment domain of the SCT, could include strategies such as placing F&V posters up in neighbourhoods [60] or F&V subscription programs in early years and school settings [61].

Recommendations for capitalizing on the power and impact of role models includes using relatable role models, humor, such as through comic books and home-based assignments to be conducted between children and their parents [5].

Strengths of this study included perspectives of a range of key informant types, such as health, school/youth, and food supply workers. In addition, sampling occurred across WA regions, degrees of remoteness, and levels of socio-economic disadvantage. This approach facilitated the inclusion of key informants who represented a range of locations with varying levels of disadvantage, and a range of work disciplines. In addition, this study had a strengths-based focus, and as such captured positive perspectives regarding potentially successful approaches to increasing rural and remote children’s F&V intake. Further, the use of the SCT is useful to investigate health behavior constructs and not only considers the impact of personal factors, such as food preferences, on behavior, but also the impact of environmental factors, such as food availability, on the behavior. This study also has some limitations that must be acknowledged. This study included the views of key informants and did not incorporate parent’s or children’s views, which were captured in another study aspect. Therefore, in relation to potentially effective strategies to increase children’s F&V consumption, a lack of child perspectives for the strategies proposed is a clear limitation. In addition, this study did not specifically focus on proposing strategies for increased F&V consumption among particular population groups, such as Culturally and Linguistically Diverse or Aboriginal and Torres Strait Islander people. Further, although attempts were made to ensure every worker type was represented across each region, this was not the case.

## 5. Conclusions

Overall, a comprehensive range of strategies has been recommended as an effective approach to increase F&V consumption among children [5,61]. Strategy components incorporating curriculum-based education programs, a focus on parental provision in the home environment, and F&V provision in other settings are essential [5,61].

## Figures and Tables

**Table 1:** Exemplar quotes for strategies to increase children’s fruit and vegetable intake.

Strategy	Exemplar Quote
Strategy One: Use relevant motivators for children to increase their preference for fruit and vegetables	*“That’s a motivator—“You look good, strong, fit, you look great”. How do you sustain that? For young women too. Playing on that strength, that useful, you’re really concerned about your appearance, playing on that strength in a positive way is a good motivator. It’s the first time I’ve really seen teenage boys engage, when it’s talking about them and their appearance”* (Health Worker)
Strategy Two: Empower local community-driven initiatives	*“Involving local people in the creation of local resources rather than depending on outside resources for health promotion”* (School and Youth Worker)
Strategy Three: Increase food literacy education across a range of settings	*“There is the occasional dietitian doing a talk for a mothers group and that sort of thing. No sort of big focus on that audience. More of a focus once kids get to school or once a child is obese and comes to see a clinician. I think prevention would be best sort of in a way, treatment for obesity in that, before a kid gets to that point”* (Health Worker)
Strategy Four: Develop salient key messages and cooking tips that resonate with parents	*“In some cases, I’ve suggested or parents have said to use the star charts, to try new things basically so they would get a star just trying something new”* (Health Worker)
Strategy Five: Increase fruit and vegetable availability, safety and convenience	*“Pre-packed salads, little salads, I also did something myself trying to increase the availability of snack packs through fruit and veg, I cut up capsicum, carrots and celery and put that in a little container. Again for somebody who wanted a little snack pack, it’s easy, it’s there, done, it’s all prepared for you but it’s just like having a bag of chips, it gives you the option”* (Food Supply Worker)
Strategy Six: Consider the impact of role models that extend beyond the family	*“The youth workers organised last year to have one of the big Western football teams Skype into all of the kids at the school... significant people being involved in people’s lives here makes a huge difference”* (School and Youth Worker)
